# Bone marrow cells are differentiated into MDSCs by BCC‐Ex through down‐regulating the expression of CXCR4 and activating STAT3 signalling pathway

**DOI:** 10.1111/jcmm.16559

**Published:** 2021-05-06

**Authors:** Quan‐Wen Liu, Yong Chen, Jing‐Yuan Li, Ling Xiao, Wen‐Jie Zhang, Jia‐Le Zhao, Hao‐Cheng Gu, Han‐You Wu, Guo‐Si‐Lang Zuo, Ke‐Yu Deng, Hong‐Bo Xin

**Affiliations:** ^1^ The National Engineering Research Center for Bioengineering Drugs and the Technologies Institute of Translational Medicine Nanchang University Nanchang China; ^2^ School of Chemistry, Biology and Material Science East China University of Technology Nanchang China; ^3^ School of Life and Science Nanchang University Nanchang China

**Keywords:** breast cancer, CXCR4, exosomes, MDSCs, STAT3

## Abstract

Studies showed that the increase of myeloid‐derived suppressor cells (MDSCs) in tumour microenvironment is closely related to the resistant treatment and poor prognosis of metastatic breast cancer. However, the effect of tumour‐derived exosomes on MDSCs and its mechanism are not clear. Here, we reported that breast cancer cells (4T1)‐secreted exosomes (BCC‐Ex) were able to differentiate bone marrow cells into MDSCs and significantly inhibited the proliferation of T lymphocytes to provide an immunosuppressive microenvironment for cancer cells in vivo and in vitro. The number of MDSCs in bone marrow and spleen of 4T1 tumour‐bearing mice and BCC‐Ex infused mice was significantly higher than that of normal mice, whereas the number of T lymphocytes in spleen was significantly decreased. In addition, BCC‐Ex markedly promoted the differentiation of MDSCs from bone marrow cells or bone marrow cells derived macrophages, seen as the increased expressions of MDSCs‐related functional proteins Arginase‐1 (*Arg‐1*) and inducible nitric oxide synthase (*iNOS*). Furthermore, BCC‐Ex significantly down‐regulated the expressions of chemokine receptor *CXCR4* and markedly up‐regulated the levels of inflammatory cytokines *IL‐6* and *IL‐10* in bone marrow cells and macrophages and remarkably inhibited the division and proliferation of T cells. Importantly, *CXCR4* agonist, *CXCL12*, could reverse the function of BCC‐Ex, indicating that BCC‐Ex‐induced MDSCs might be dependent on the down‐regulation of *CXCR4*. Western blot showed that BCC‐Ex significantly promoted the phosphorylation of *STAT3* in bone marrow cells, resulting in the inhibitions of the proliferation and apoptosis of bone marrow cells, and the aggravation of the differentiation of bone marrow cells into MDSCs.

## 1 INTRODUCTION

Breast cancer is one of the most common malignant tumours in women with a incidence about 1/8‐1/10.^1^, ^2^ At present, the treatments of breast cancer include surgery, chemotherapy, radiotherapy, endocrine therapy and molecular targeted therapy.^3^, ^4^ In the case of highly invasive tumours such as triple‐negative breast cancer which is easy to relapse and metastasize in the later stage, the general treatment could not achieve a good result.^5^ Numerous clinical studies confirmed that there was an important relationship between the insensitivity of late metastatic patients to immunotherapy and the complex tumour microenvironment.^6^ Myeloid‐derived suppressor cells (MDSCs) are important components of the immunosuppressive microenvironment in tumours. The increase in MDSCs is closely associated with the resistant treatment and poor prognosis of metastatic breast cancer.^7^

Normally, myeloid hematopoietic stem cells differentiate into immature bone marrow cells, which migrate to different peripheral organs and then further differentiate into macrophages, dendritic cells or neutrophils.^8^ However, in the case of acute or chronic inflammation, infectious trauma and tumour, an accumulation of the immature bone marrow cells will occur, thus resulting in immunosuppression, locally.^8^, ^9^ In mice, MDSCs express both myeloid differentiation antigens *Gr‐1* and *CD11b* ^10^ and the cells are further defined as monocytic MDSCs (mMDSCs) and granulocytic MDSCs (gMDSCs), in which the mMDSCs and gMDACs are characterized with *CD11b+Ly6G‐Ly6C^high^* and *CD11b+Ly6G+Ly6C^low^* phenotypes, respectively.^11^, ^12^ In general, MDSCs produce immunosuppression by directly or indirectly influencing a variety of immune cells such as inhibiting the proliferation of T cells, destroying the function of NK cells, and inducing macrophages to differentiate into M2 types and so on.^13^, ^14^, ^15^

Exosome, a lipid bilayer structure with a diameter of about 50‐200 nm, plays an important role in the intercellular communication and tumour microenvironment reprogramming.^16^ As a potential biomarker and therapeutic target of tumour, exosome has received extensive attention in recent years.^17^, ^18^ Studies showed that tumour cells‐derived exosomes could be secreted into the microenvironment of tumour, which is crucial to the invasion and metastasis of tumour cells and the proliferation and survival of immune cells.^19^ There were studies showing that tumour exosomes promoted the production of *IL‐6* and inhibited the differentiation of the *CD11b+* myeloid progenitor cells into dendritic cells and macrophages in bone marrow, thus promoting tumour growth.^20^ In addition, exosomes derived from tumour cells could be able to make MDSCs secrete inflammatory cytokines such as *IL‐6* and *TNF‐α*, which further promoted the expansion of MDSCs. The continuous expansion of MDSCs around the breast cancers could further lead to tumour deterioration and immune escape.^21^ However, the molecular mechanism of the tumour‐derived exosomes in induction of inflammatory factors and the production of MDSCs is not clear.

Signal transduction and activator of transcription 3 *(STAT3)* is a transcription factor of the STAT family, which is highly expressed in many malignant tumours. The activation of *STAT3* led to abnormal cell proliferation and malignant transformation.^22^ Studies showed that breast cancer cell‐derived exosomes (BCC‐Ex) promoted the production of MDSCs in bone marrow to induce immunosuppression through activating *STAT3* signalling pathway,^23^ thus promoting the invasion and metastasis of breast cancer.^24^ The chemokine receptor *CXCR4* and its ligand *CXCL12* play an important role in the immune and inflammatory response, invasion and metastasis of malignant tumours, and stem cell migration and homing. Goloviznina et al^25^ observed that tumour exosomes significantly down‐regulated the expression of *CXCR4* on mouse bone marrow cells, resulting in a decrease in their ability to migrate along the *CXCL12* gradient.

In the present study, an accumulation of a large amount of MDSCs in bone marrow and spleen and a significant reduction of the killer T lymphocytes in spleen were observed in 4T1 tumour‐bearing mice compared with normal mice, suggesting the formation of immunosuppressive microenvironment. Further studies showed that the BCC‐Ex differentiated the bone marrow cells into MDSCs and inhibited T cells proliferation by reducing the expression of *CXCR4* in bone marrow cells. Furthermore, BCC‐Ex also inhibited the proliferation and apoptosis of bone marrow cells and stimulated the differentiation of bone marrow cells into MDSCs by activating *STAT3* signal pathway.

## 2 MATERIALS AND METHODS

### 2.1 Animal models

BALB/c mice were obtained from Changsha SLAC Laboratory Animal Company (http://www.hnsja.com/). All animal experiments were performed according to institutional guidelines and approved by the Animal Care and Use Committee of Nanchang University.

### 2.2 Isolation of mouse bone marrow cells

Kill Balb/c mice by cervical dislocation and spray whole body thoroughly with 70% ethanol solution. Carefully take out the femur and tibia and place them in a tube of ice‐cold, sterile PBS. Prepare a syringe full of 37°C medium (RPMI 1640 (Gibco), supplemented with 10% FBS (Gibco)), with a 27‐gauge needle and a sterile 50 mL tube (Sorfa). Cut the top and bottom ends of the femur and tibia with sterile scissors. Insert the needle through the cut end and flush bone marrow with medium into a sterile tube. Blow the bone marrow into single cell suspension. The single cell suspension was filtered through a 70‐μm cell strainer (BD Labware) and centrifuge at 1000 rpm for 5 minutes. The supernatant was discarded, and the cells were resuspended with RPMI medium supplemented with 10% FBS. Bone marrow cells were placed in cell culture dishes at 37°C, with 5% CO_2_ atmosphere.

### 2.3 Isolation of mouse spleen cells

Aseptically remove spleen and place it in a tube of ice‐cold, sterile PBS. Remove any remaining fat and connective tissue using two needles and transfer spleen to a small petri dish containing 7 mL enzyme digestion mix (RPMI 1640, supplemented with collagenase type III at 1 μg/mL and Dnase I at 20 μg/mL). Cut the tissue into very small fragments, digesting the tissue for 20‐25 minutes at room temperature. Add 600 μL of EDTA solution to the digestion mix and continue the incubation for a further 5 minutes. Run the digestion mix through a 70‐μm cell strainer to remove any remaining undigested tissue. The cells were treated with aseptic erythrocyte lysate for 5 minutes at room temperature and then add 10 mL precooled PBS to terminate the lysis. The single cell suspension was centrifuged at 1000 rpm for 5 minutes and discarded the supernatant, and the cells were resuspended with RPMI medium supplemented with 10% FBS.

### 2.4 Flow cytometry

Cells were digested by 0.05% trypsin‐EDTA (Gibco), and cell suspension were filtered using 200‐mesh filter. After 5 minutes of centrifugation, supernatant was removed and cells were resuspended with stain FACS buffer (PBS containing 2% FBS) at a concentration of 1 × 10^6^ cells/mL. The cells then incubating with primary antibodies (*CD11b*‐FITC, *Ly6C*‐PE, *CD3*‐FITC, *CD8*‐APC, *CXCR4*‐PE, *CX3CR1*‐APC, *Gr‐1*‐APC) and their isotype controls (all from BD Biosciences)at 4°C for 30 minutes in the dark. After washing twice, the cells were resuspended in 200 μL of PBS and acquired by a FACSCalibur instrument (BD Biosciences). Data were analysed using FLOWJO TM software (TreeStar, Inc.).

### 2.5 Isolation of exosomes

When the breast cancer cells (4T1) were reached to 80%‐90% confluent the cells were washed with PBS and cultured for an additional 2 days in 4T1 cell medium with exosome‐free FBS. The conditional medium was collected and centrifuged at 500 *g* for 5 minutes at 4°C to remove the dead cells, cell fragments and apoptotic bodies. After the centrifugation, the supernatant was transferred to an Ultra‐clear tube (Millipore) and centrifuged at 100 000 *g* for 70 minutes at 4°C to deposit BCC‐Ex, and then the supernatant was decanted. The BCC‐Ex pellet was resuspended in 200 μL of PBS and stored at −80°C or used for other downstream experiments. The concentrations of BCC‐Ex protein were determined using the BCA (Thermo) assay following the instructions (Thermo Fisher). The absorbance was read at 562 nm using a Microplate Reader (Bio‐Rad Laboratories).

### 2.6 Transmission electron microscopy

The morphology and size distribution of exosomes were identified by using transmission electron microscopy. Briefly, the exosomes were fixed in 3% glutaraldehyde and 2% paraformaldehyde in cacodylate buffer and then loaded to formvar‐carbon coated copper grids. After washing, the grids were contrasted in 2% uranyl acetate, dried and then examined by TEM (JEM‐1400PLUS).

### 2.7 Western blot analysis

Exosomes and total protein were run on 10% denaturing SDS‐PAGE gels, then transferred to nitrocellulose membranes (BioRad), which were incubated with primary antibodies anti‐*GAPDH* (1:1000, rabbit monoclonal, Santa Cruz), anti‐*CD9* (1:2000, rabbit monoclonal, Abcam), anti‐*Rab5* (1:1000, rabbit monoclonal, Abcam), anti‐*TSG101* (1:1000, rabbit monoclonal, Abcam), *β‐actin* (1:1000, mouse polyclonal, CST), *CXCR‐4* (1:100, rabbit monoclonal, Abcam), *IL‐6* (1:1000, rabbit monoclonal, Abcam), *IL‐10* (1:1000, rabbit monoclonal, Abcam), *Arg‐1*(1:1000, rabbit monoclonal, CST), *iNOS* (1:1000, rabbit monoclonal, Abcam), *STAT3* (1:1000, mouse monoclonal, CST), Phospho‐*STAT3* (1:2000, rabbit monoclonal, CST), *Bax* (1:1000, mouse monoclonal, Abcam), *PCNA* (1:1000, mouse monoclonal, Abcam) at 4°C overnight. Blots were detected with horseradish peroxidase (HRP)‐conjugated goat anti‐rabbit or rabbit anti‐mouse secondary antibody (Invitrogen) for 1 hour at room temperature. Images were quantified using the Super Signal West Pico or Femto chemiluminescent detection system (Pierce).

### 2.8 Maturation, polarization and BCC‐Ex treatment of bone marrow‐derived macrophages

Maturation of macrophages: On the first day, primary bone marrow cells were cultured in cell culture dishes, and M‐CSF (25 ng/mL) were added into the medium at the same time. At day 3, half of the medium was changed, 25 ng/mL M‐CSF were added. At day 5, the medium was fully changed, 25 ng/mL M‐CSF were added. The bone marrow cells differentiated into M0 type macrophages.

Macrophage polarization: At day 6, add 10 ng/mL IL‐4 to stimulate macrophages to M2a polarization. At day 7, add 10 ng/mL LPS and 10 ng/mL IFN‐γ to stimulate macrophages to M1 polarization, and add 10 ng/mL IL‐10L to stimulate macrophages to M2C polarization.

Exosome treatment: M0, M1, M2a, M2C macrophages were treated with BCC‐Ex at the concentration of 30 μg/mL, for 24 hours.

### 2.9 Microarray

After M0 macrophages were treated with PBS or BCC‐Ex for 24 hours, the RNA of M0 macrophages and BCC‐Ex treated M0 macrophages were extracted by TRIzol and detected by gene chip (Agilent SurePrint G3 Mouse GE V2.0, 8*60K, Design ID:074809).

### 2.10 Quantitative real‐time PCR

Total RNA from the cells was extracted using the RNA isolation kit (Agilent technologies) per the manufacturer's instructions. After removing the genomic DNA using DNase I (Ambion), 1 μg RNA was reverse‐transcribed into cDNA using a commercially available kit (Applied Biosystems). Quantitative real‐time PCR was performed with iCycler Thermal Cycler (Bio‐Rad) using 2X SYBR Green master mix (Bio‐Rad). Forty cycles were conducted as follows: 95°C for 30 seconds, 60°C for 30 seconds, preceded by 1 minute at 95°C for polymerase activation. Primer sequences for all genes we measured in this report are shown in Table 1. Quantification was performed by the delta cycle time method, with *GAPDH* used for normalization.

**TABLE 1:** Primers for qPCR

Genes		Sequence
GAPDH	F	ACCCAGAAGACTGTGGATGG
	R	ACACATTGGGGGTAGGAACA
CX3CR1	F	CTGTTATTTGGGCGACATTG
	R	AACAGATTTCCCACCAGACC
CXCR4	F	CCGGTACCTCGCTATTGTCC
	R	TCCACAGGCTATCGGGGTAA
IL‐10	F	AGACACCTTGGTCTTGGAGC
	R	TTTGAATTCCCTGGGTGAGA
IL‐6	F	TGGTACTCCAGAAGACCAGAGG
	R	AACGATGATGCACTTGCAGA
Arg‐1	F	TTTTCCAGCAGACCAGCTTT
	R	GGAACCCAGAGAGAGCATGA
iNOS	F	CCTTGGTGAAGGGACTGAGC
	R	TGCTGTGCTACAGTTCCGAG

Abbreviations: F, Forward primer; R, Reverse primer.

### 2.11 Sorting of CD3 positive T cells from spleen cells by magnetic beads

Resuspend the spleen cells in MACS™ buffer in a 15 mL centrifugation tube. Add CD3 + MACS™ beads according to the manufacturer's instructions and vortex. Incubate at 4°C for 15 minutes. Fill tube to 15 mL with MACS™ buffer, invert 3 times and centrifuge for 10 minutes, 4°C, 300 *g*. Discard supernatant and resuspend the cell pellet in MACS™ buffer. Purify CD3+ T cells using a MACS™ MS column in a Mini MACS™ magnet.

### 2.12 CFSE labelling CD3 positive T cells

*CD3*‐positive T cells were suspended with 1 mL RPMI medium without serum, and CFSE (5 μmol/L) was added. After incubating 20 minutes in 37°C incubator, the complete culture medium was added and placed it on ice for 5 minutes to stop the reaction and then, the sample was centrifugated at 4°C, 225 g for 5 minutes, and the supernatant was discarded. After washing twice with 5 mL complete culture medium, the CSFE‐labelled *CD3+* T cells were placed in cell culture dishes at 37°C, with 5% CO_2_ atmosphere.

### 2.13 Statistical analysis

The results are presented as average value ± standard deviation (SD). Student's *t* test was used for analysis between two groups. One‐way analysis of variance (ANOVA) was used to compare data among three or more groups. Differences with a *P*‐value of < .05 were considered statistically significant.

## 3 RESULTS

### 3.1 Myeloid‐derived suppressor cells (MDSCs) were accumulated in bone marrow and spleen and T lymphocytes were reduced in spleen of 4T1 breast cancer‐bearing mice

**FIGURE 1:**
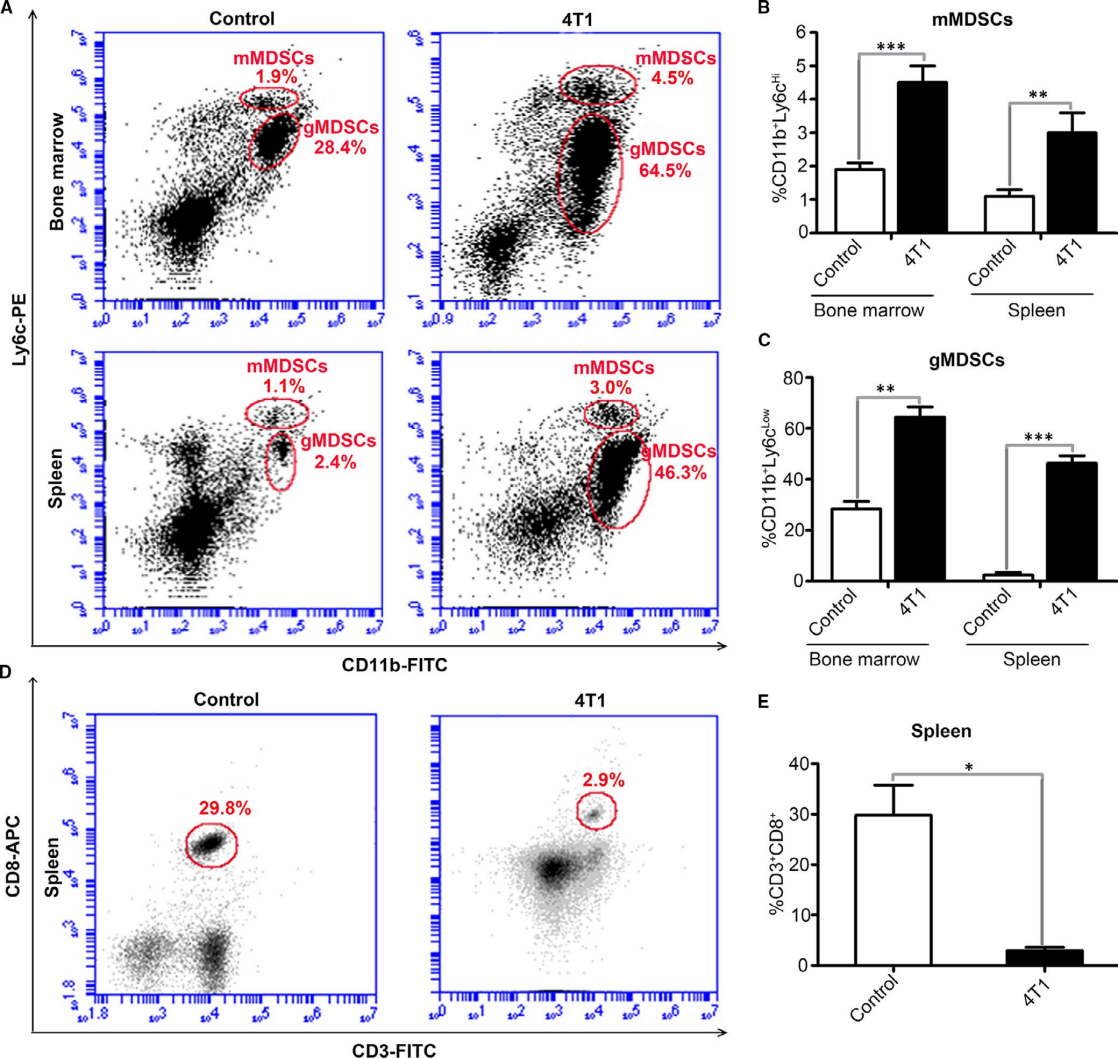
The amount changes of mMDSCs, gMDSCs and cytotoxic T lymphocytes in bone marrow and spleen of normal control mice and 4T1 tumour‐bearing mice. A, Detect the amounts of mMDSCs and gMDSCs in bone marrow and spleen of normal mice and 4T1 tumour‐bearing mice by flow cytometry. B, Quantitative analysis of the amounts of mMDSCs in bone marrow and spleen of normal mice and 4T1 tumour‐bearing mice in figure A (n = 3). C, Quantitative analysis of the amounts of gMDSCs in bone marrow and spleen of normal mice and 4T1 tumour‐bearing mice in figure A (n = 3). D, The proportion of CD3 + CD8 + T lymphocytes in the spleen of normal mice and 4T1 tumour‐bearing mice was detected by flow cytometry. E, Quantitative analysis of the proportion of CD3+CD8+ in the spleen of normal mice and 4T1 tumour‐bearing mice in D figure (n = 3). Significance was measured using a two‐way ANOVA. *P < .05, **P < .01, ***P < .001

A total of 4 × 10^5^ 4T1 breast cancer cells were injected into the breast fat pad of the 8‐week‐old Balb/c mice to establish a model of breast cancer. After 4 weeks, the palpable tumours were observed in the injection sites (Figure S1). Fresh bone marrow cells and spleen cells were isolated, and the expressions of *CD11b* and *Ly6C* were detected by flow cytometry analysis. The results showed that the number of mMDSCs (*CD11b+Ly6C^high^*) and gMDSCs (*CD11b+Ly6C^low^*) in bone marrow and spleen of 4T1 tumour‐bearing mice were significantly increased compared with normal mice. However, the number of mMDSCs in bone marrow and spleen was obviously less than that of gMDSCs (Figure 1A‐C). These results indicated that there was a large amount of MDSCs accumulation in the bone marrow and spleen of 4T1 breast cancer model mice. In order to confirm the effect of the exosomes from breast cancer cell on MDSCs differentiation, we then established the mouse model of breast cancer with E0771 and MAD‐MB‐231 cells. The numbers of mMDSCs and gMDSCs in bone marrow and spleen in the tumour‐bearing mice with breast cancer cell lines E0771 and MAD‐MB‐231 were also significantly increased compared with normal mice (Figure S2).

The increase in *CD8 +* T lymphocyte level is positively correlated with the good prognosis of breast cancer and other cancers. Flow cytometry showed that the number of *CD3 + CD8 +* cells in the spleen of normal mice was about 29.8%, whereas the cells in the spleen of 4T1 tumour‐bearing mice were decreased to 2.9% (Figure 1D,E), indicating that the reduced killer T lymphocytes in spleen of 4T1 tumour‐bearing mice might be benefit to the formation of immunosuppressive microenvironment.

### 3.2 The polarizations of macrophages were altered by breast cancer cell‐derived exosomes (BBC‐Ex)

**FIGURE 2:**
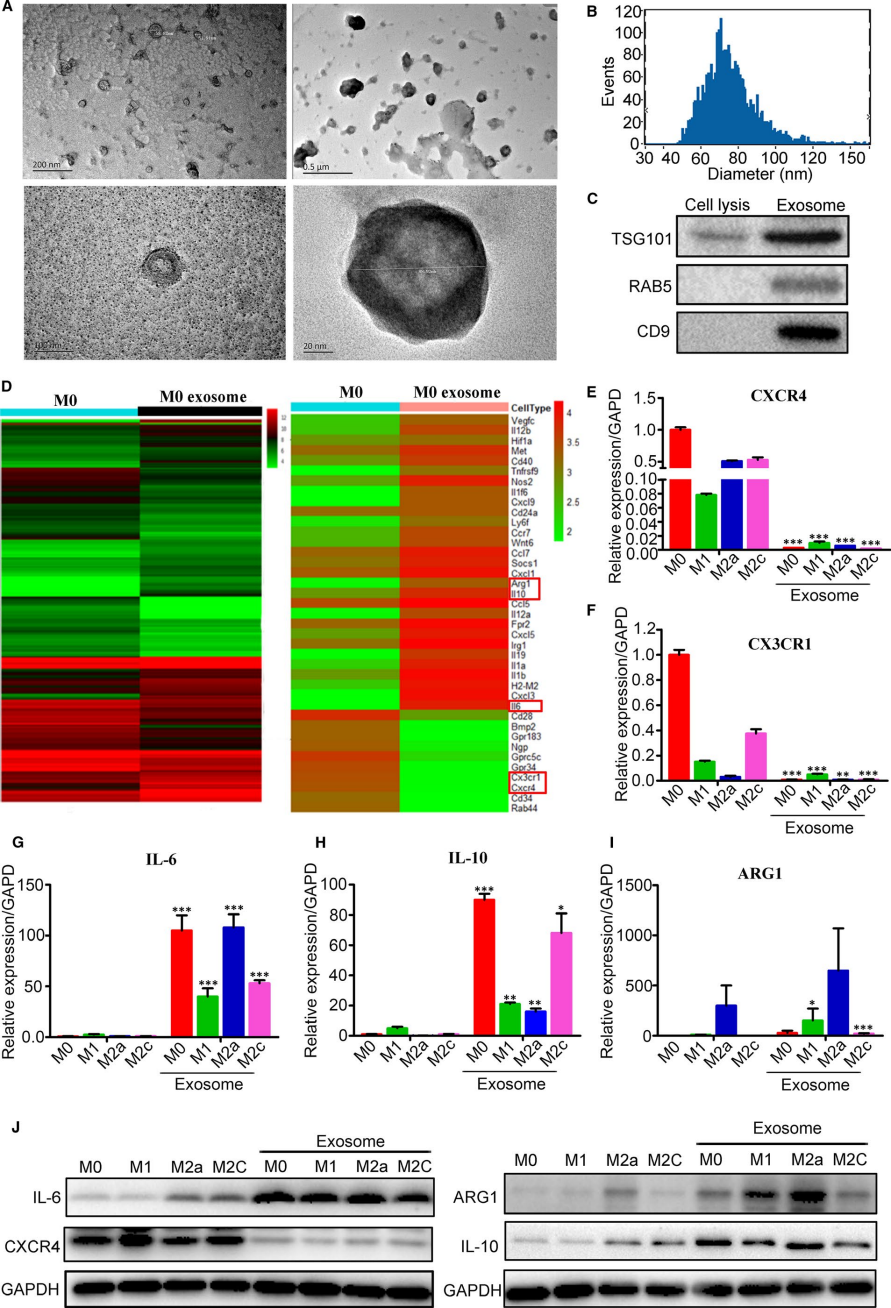
Effect of 4T1 breast cancer cells derived exosomes on the polarization of bone marrow‐derived macrophages. A, The morphology of exosomes was detected by transmission electron microscope. B, Size distribution analysis of BCC‐Ex was detected by NTA. C, The expression of exosomes associated surface marker protein TSG101, RAB5 and CD9 was detected by Western blot. D, The changes of gene expression in MO type macrophages and BCC‐Ex treated MO type macrophages were detected by microarray method. Left: all gene changes. Right: differentially expressed gene heat map. E‐I, The results of microarray were verified by qPCR, and the genes detected were CXCR4, CX3CR1, IL‐6, IL‐10, Arg‐1 (n = 3). J, The results of microarray were verified by Western blot, and the genes detected were CXCR4, IL‐6, IL‐10 and Arg‐1. Significance was measured using a two‐way ANOVA. *P < .05, **P < .01, ***P < .001 vs control

Various immune cells are involved in tumour immunosuppressive microenvironment, among which the tumour‐associated macrophages account for 50% of the total number of these cells. Tumour cells secrete a large number of exosomes, which play an important role in the tumour microenvironment. In order to explore the effect of tumour cells‐secreted exosomes on macrophages, the 4T1 BBC‐Ex were isolated by ultra‐high speed centrifugation. Transmission electron microscopy and nanoparticle tracking analysis (NTA) showed that the exosomes were round or oval cystic vesicles with an about 30‐150 nm diameter of a lipid bilayer structure, which were accorded with the morphological characteristics of exosomes (Figure 2A,B). Western blot showed the exosome marker proteins *CD9*, *RAB5* and *TSG101* were highly expressed in the exosomes, whereas only TSG101 protein could be detected in the cell lysate (Figure 2C), confirming that the extracts were exosomes. The differential expressions of the genes in M0 type macrophages treated with or without BBC‐Ex (30 μg/mL) were determined by gene chip sequencing (Figure 2D). The results showed that the expressions of chemokine receptors *CXCR4* and *CX3CR1* were significantly down‐regulated by BCC‐Ex in the macrophages, suggesting that the chemotaxis of macrophages was decreased by the exosomes. Our results also showed that the expressions of the inflammation‐related genes such as *IL‐6*, *IL‐10* and *Arg‐1* were significantly up‐regulated by BCC‐Ex in the macrophages. In addition, BCC‐Ex‐induced the reduction in the expression of co‐stimulatory molecule *CD28* in M0 type macrophages suggested a decrease in immunoreactivity of macrophages. The alternations of these genes were quantitatively confirmed by qPCR assay and Western blot (Figure 2E‐J), suggesting that the myeloid‐derived macrophages could be polarized by BCC‐Ex into various types of the macrophages. These results indicated that the BCC‐Ex promoted inflammation by altering the polarization, chemotaxis and migration ability of the macrophages.

### 3.3 BCC‐Ex differentiated bone marrow cells into MDSCs

**FIGURE 3:**
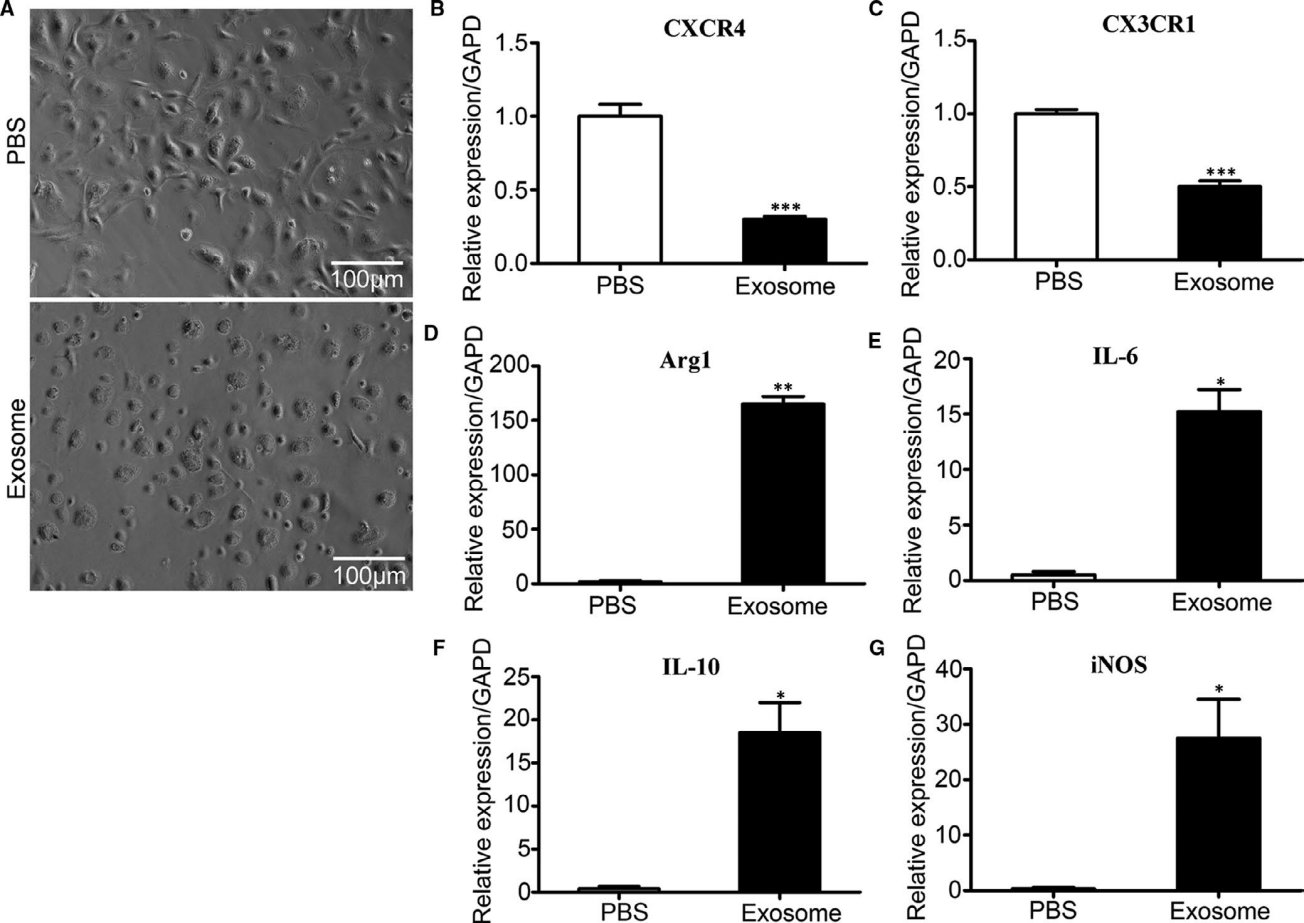
Changes in the expression of chemokine receptors and MDSCs‐related genes in bone marrow cells and BCC‐Ex treated bone marrow cells. A, Morphology of bone marrow cells after treatment of PBS and BCC‐Ex. B and C, The mRNA levels of chemokine receptor CXCR4 and CX3CR1 in PBS treated bone marrow cells and BCC‐Ex treated bone marrow cells were detected by qPCR. D‐G, The mRNA levels of MDSCs immunosuppression related genes Arg‐1, IL‐6, IL‐10 and iNOS in PBS treated bone marrow cells and BCC‐Ex treated bone marrow cells were detected by qPCR. Significance was measured using a two‐way ANOVA. *P < .05, **P < .01, ***P < .001

**FIGURE 4:**
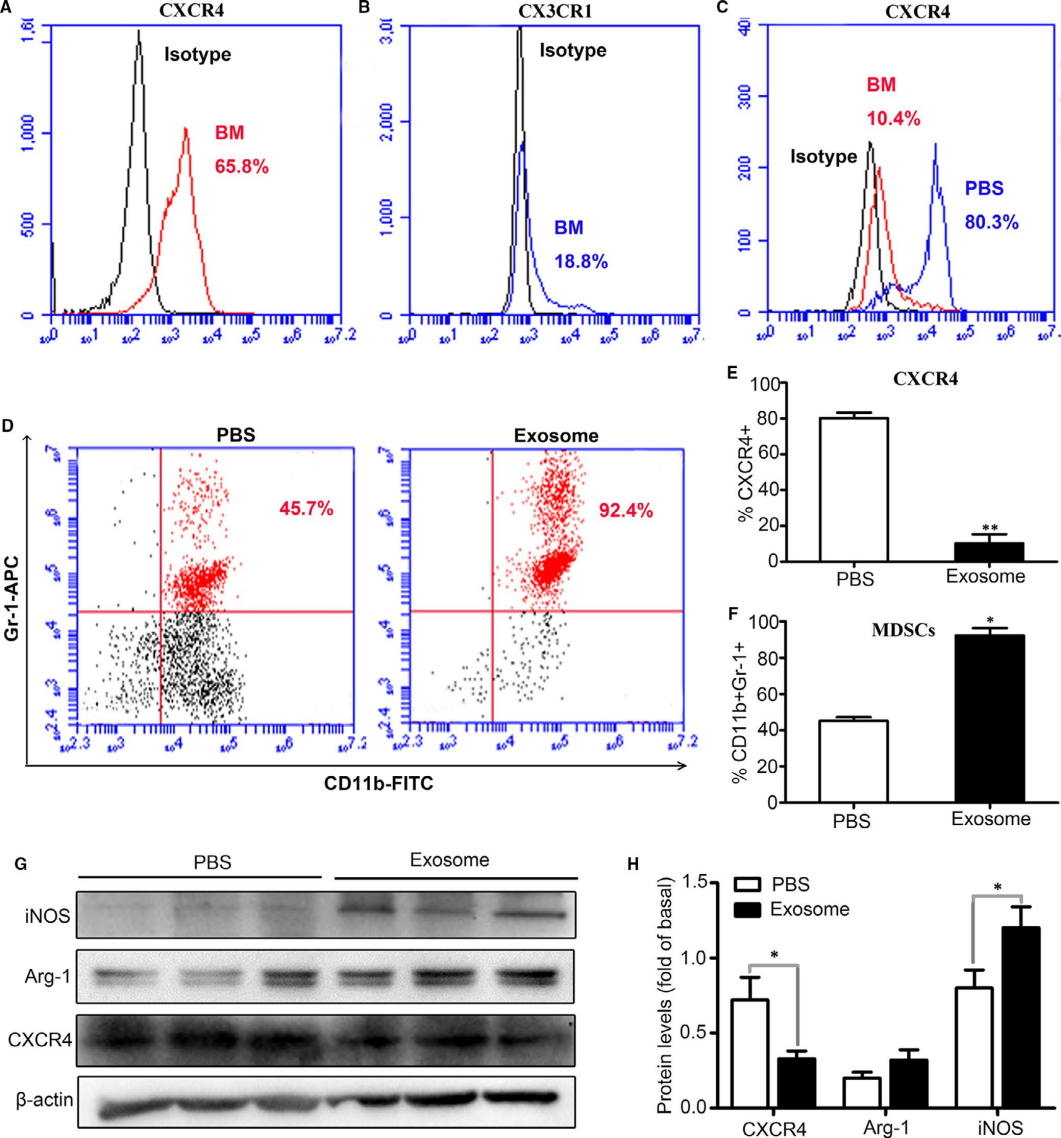
BCC‐Ex induced bone marrow cells differentiate into MDSCs. A and B, The expression levels of CXCR4 and CX3CR1 in bone marrow cells were detected by flow cytometry. C, The expression levels of CXCR4 in PBS and BCC‐Ex treated bone marrow cells were detected by flow cytometry. D, Examination of MDSCs (CD11b+Gr1+) in PBS and BCC‐Ex treated bone marrow cells by flow cytometry. E, Quantitative Analysis of the percentage of CXCR4‐positive cells as shown in C. F, Quantitative Analysis of the percentage of MDSCs as shown in D. G, The expression analysis of CXCR4, Arg‐1 and iNOS in bone marrow cells treated with PBS and 4T1 BCC‐Ex by immunoblotting assay. H, Quantitative Analysis of data of figure G. Significance was measured using a two‐way ANOVA. *P < .05, **P < .01

After stimulated with M‐CSF (50 ng/mL) for 24 hours, the primary bone marrow cells were treated with exosomes derived from 4T1 cells (30 μg/mL) and PBS treatment group was used as a control. The results showed that after stimulation with M‐CSF, the bone marrow cells in the control group exhibited a long spindle‐shaped, whereas the bone marrow cells in the BCC‐Ex treatment group were round and irregular (Figure 3A). Our results also showed that BCC‐Ex significantly reduced the expressions of *CXCR4* and *CX3CR1* and elevated the mRNA levels of *Arg‐1*, *IL‐6*, *IL‐10* and *iNOS,* which was related to MDSCs immunosuppression (Figure 3B‐G), suggesting that the exosomes released from breast cancer cells down‐regulated the expression of chemokine receptors in bone marrow cells, up‐regulated the expression of genes related to MDSCs immunosuppression, and differentiated bone marrow cells into MDSCs. Flow cytometry results showed that there were a high expression of *CXCR4* (65.8%, Figure 4A) and a low expression of *CX3CR1* (18.8%, Figure 4B) in bone marrow cells, suggesting that *CXCR4* might be responsible for the differentiation of bone marrow cells into MDSCs induced by BCC‐Ex. The expression of *CXCR4* in bone marrow cells was decreased from 80.3% to 10.4% (Figure 4C,E) in BCC‐Ex treatment group compared with control group, whereas MDSCs (*CD11b+Gr1+*) were increased from 45.2% to 92.4% (Figure 4D,F). MDSCs‐derived *Arg‐1* and *iNOS* could induce apoptosis of T cells. The results from Western blot analysis were consistent with the alternations of the mRNA, in which the CXCR4 levels were decreased and the *Arg*‐1 and *iNOS* levels were increased by BCC‐Ex stimulation (Figure 4G,H). The above results demonstrated that the BCC‐Ex promoted the differentiation of bone marrow cells into MDSCs through reducing the expression of CXCR4 and increasing the expressions of the proteins related to MDSCs immunosuppressive function in bone marrow cells.

### 3.4 Bone marrow cells treated with BCC‐Ex inhibited the proliferation of T lymphocytes

**FIGURE 5:**
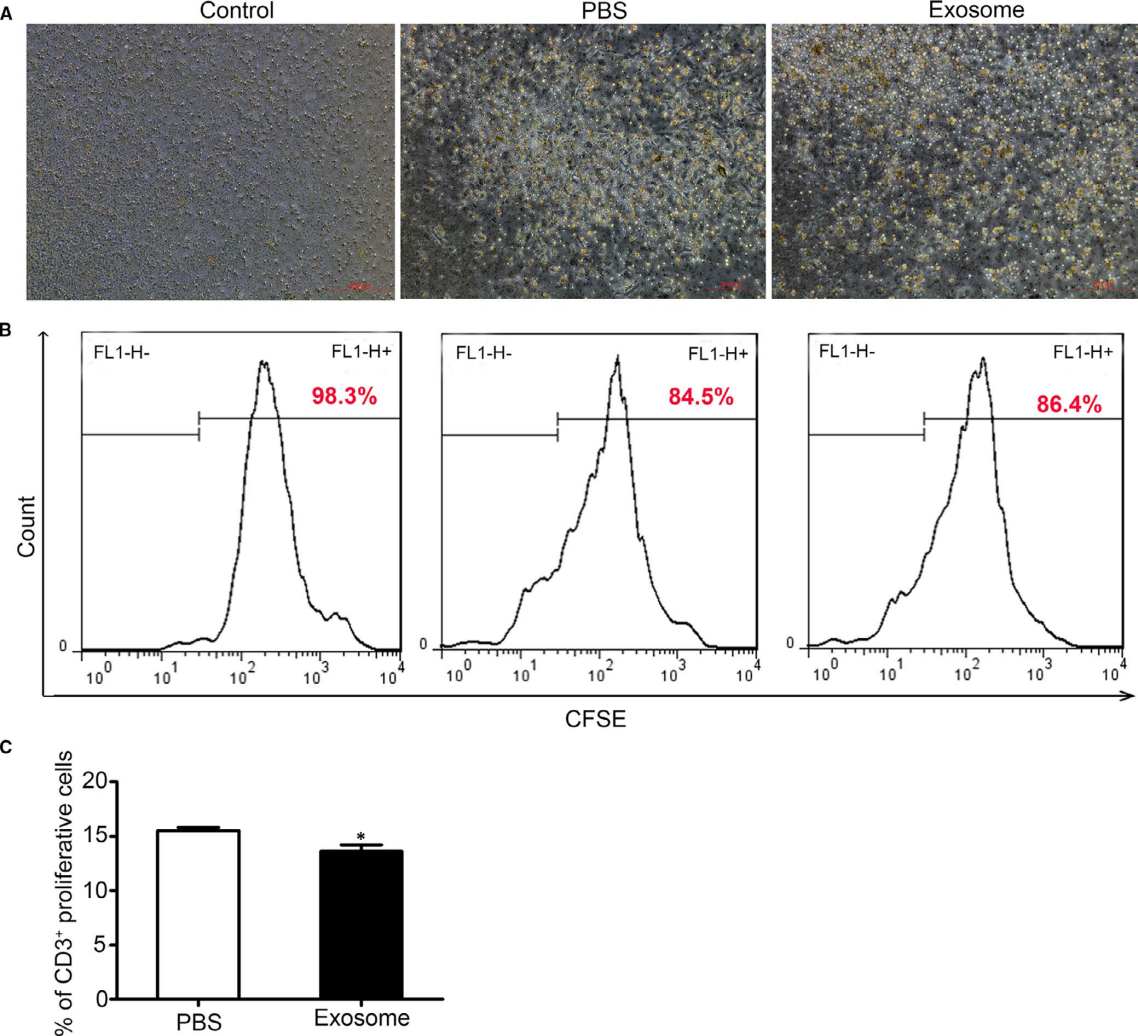
The bone marrow cells treated by BCC‐Ex have the function of inhibiting T cell proliferation and activation. A, Representative images of the T cells proliferation in control, PBS and exosome group. B, The proportion of CEFS positive cells was detected by flow cytometry. The peak shift to the left indicates the division and proliferation of T cells. C, The proportion of CD3+ cells in the proliferative phase in PBS group and exosome group. Significance was measured using a two‐way ANOVA. *P < .05

CFSE, a fluorescent probe, is widely used to detect cell proliferation in recent years, and it is also used for tracing of cell fluorescence. T lymphocytes (T cells) from the spleen of Balb/c mice were sorted with *CD3* positive sorting kit and labelled with CFSE. After treated with BCC‐Ex for 24 hours, the bone marrow cells were co‐cultured with T cells and then, T‐cell proliferation was stimulated by anti‐CD3 (1 μg/mL) and anti‐CD28 (2 μg/mL) antibody (Exosome group). After 72 hours, T cells were collected and the fluorescence intensity of CFSE was detected by flow cytometry. The results showed the BCC‐Ex decreased the numbers of T cells (Figure 5A) and increased the CFSE positive cells (Figure 5B). In addition, the *CD3+* cells in the proliferative phase were decreased from 15.5% to 13.6% (Figure 5C). The above results indicated that the bone marrow cells treated by 4T1‐Ex were able to inhibit T‐cell proliferation, in which the inhibition was consistent with MDSCs.

### 3.5 BCC‐Ex promoted MDSCs induction and inhibited T lymphocyte proliferation in vivo

**FIGURE 6:**
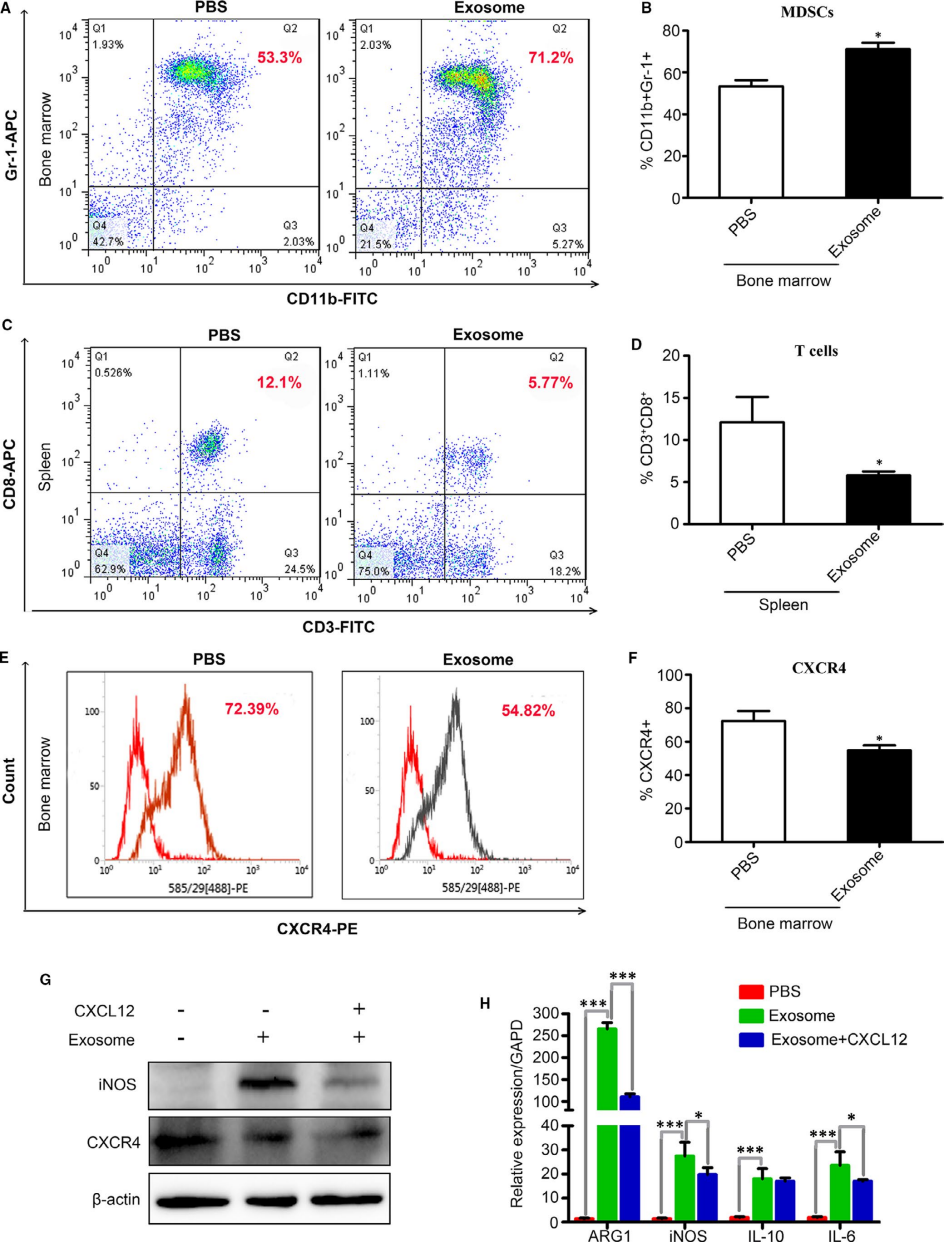
BCC‐Ex suppresses the expression of CXCR4 in bone marrow cells, promotes the accumulation of MDSCs in bone marrow and decreases the number of T lymphocytes in spleen in vivo. A, The proportion MDSCs (CD11b+Gr‐1+) in bone marrow cells of PBS injected mice and BCC‐Ex injected mice was detected by flow cytometry. B, Quantitative analysis of the percentage of MDSCs as shown in A. C, Flow cytometry was used to detect the number of T cells (CD3+CD8+) in the spleen of PBS injected mice and BCC‐Ex injected mice. D, Quantitative analysis of the number of T cells as shown in C. E, The expression level of CXCR4 in bone marrow cells of PBS injected mice and BCC‐Ex injected mice was detected by flow cytometry. F, Quantitative analysis of the corresponding data of figure E (n = 3). G, Western blot assay for CXCR4 and iNOS in bone marrow cells treated with BCC‐Ex in the presence or absence of CXCL12 (100 ng/mL). Bone marrows not treated with BCC‐Ex and CXCL12 and cultured in normal medium were used as control. H, qPCR was used to detect the expression level of mRNA of Arg‐1, iNOS, IL‐10 and IL‐6 in different groups. Significance was measured using a two‐way ANOVA. *P < .05, ***P < .01, ***P < .001

The healthy Balb/c mice were injected with 200 μL PBS containing 100 μg BCC‐Ex or 200 μL PBS through tail vein for one time. After 24 hours, the bone marrow cells and spleen cells were isolated from the above mice for flow cytometry analysis. The results showed that the numbers of MDSCs (*CD11b+Gr‐1+*) in bone marrow of mice injected with BCC‐Ex were significantly higher than that of PBS group (Figure 6A,B). The spleen of the BCC‐Ex group was slightly enlarged than that of the PBS group, and the MDSCs in the spleen were increased slightly (the data did not show), the T lymphocytes (*CD3+CD8+*) were significantly decreased (Figure 6C,D). These results indicated that BCC‐Ex differentiated bone marrow cells into MDSCs in vivo and then inhibit the proliferation and activation of T lymphocytes, providing an immunosuppressive microenvironment for breast cancers. In addition, the expressions of *CXCR4* in bone marrow were significantly decreased in exosome group (Figure 6E,F), suggesting that the effect might be the reason for the accumulation of MDSCs in bone marrow.

The role of *CXCL12/CXCR4* signal axis is to chemotaxis cell migration. In order to further clarify whether the production of MDSCs was due to the decreased chemotaxis of bone marrow cells, the bone marrow cells were treated with BCC‐Ex (30 μg/mL) with or without *CXCL12* (agonist, 100 ng/mL) for 24 hours. Western blot detection showed BCC‐Ex could decrease the expressions of CXCR4 and increase the expression of iNOS in bone marrow cells, and the effect of BCC‐Ex on iNOS could be reversed by *CXCL12* (Figure 6G). QPCR analysis shown that BCC‐Ex increased the expressions of *IL‐6* and *IL‐10* in bone marrow cells, whereas *CXCL12* reversed this effect to some extent, suggesting that BCC‐Ex‐induced the differentiation of bone marrow cells into MDSCs was partially related to the down‐regulation of *CXCR4*.

### 3.6 BCC‐Ex inhibited proliferation and apoptosis of bone marrow cells by activating STAT3 signalling pathway

**FIGURE 7:**
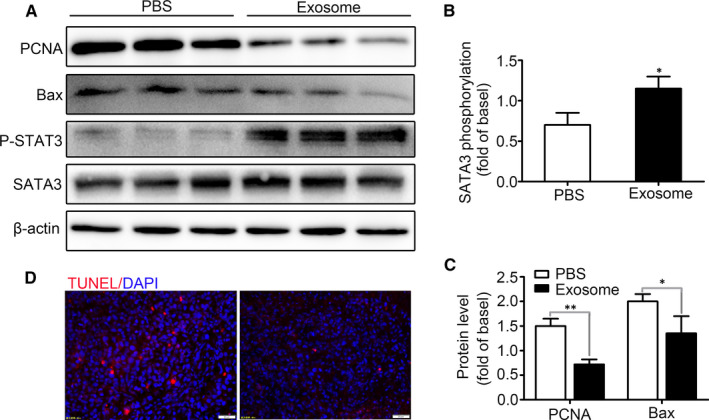
BCC‐Ex can inhibit the proliferation and apoptosis of bone marrow cells by activating STAT3 signal pathway. A, Western blot assay for SATA3, phospho‐SATA3, Bax and PCNA in bone marrows treated with PBS and BCC‐Ex. B, Quantitative analysis of the expression of p‐STAT3 in bone marrow cells of PBS group and BCC‐Ex group (n = 3). C, Quantitative analysis of the expression of Bax and PCNA in bone marrow cells of PBS group and BCC‐Ex group (n = 3). D, The apoptosis of bone marrow cells was tested by TUNEL staining. Significance was measured using a two‐way ANOVA. *P < .05, ***P < .01

STAT3 is a main regulator of MDSCs proliferation and activation. The primary bone marrow cells were treated with PBS or BCC‐Ex (30 μg/mL) for 24 hours. As shown in Figure 7A‐C, BCC‐Ex‐induced the phosphorylation of STAT3 protein was significantly increased, whereas the Bax and PCNA proteins were reduced in MDSCs. TUNEL assay showed that BCC‐Ex treatment significantly increased TUNEL‐positive cells (Figure 7D). These results indicated that BCC‐Ex could inhibit the proliferation and apoptosis of bone marrow cells and promote the differentiation of bone marrow cells into MDSCs through activating *STAT3* signalling pathway.

## 4 DISCUSSION

In recent years, the treatment of breast cancer has been continuously developed and updated, from traditional surgery, radiotherapy, chemotherapy to molecular targeted therapy, tumour immunotherapy and so on, which have significantly improved the comprehensive treatment effect and quality of life of patients. However, tumours such as triple‐negative breast cancer have a complex tumour microenvironment, and the infiltration of immature immune cells leads to drug immune tolerance, which reduces the effect of immunotherapy, making these tumours highly invasive and poor prognosis. At present, there is a lack of clear molecular therapy targets. Numerous clinical studies have shown that MDSCs in tumour microenvironment is an important component of tumour immunosuppression, and the increase in MDSCs level is closely related to the resistant treatment and poor prognosis of metastatic breast cancer.^7^ Defining the key factors affecting the production and proliferation of MDSCs and selectively targeting the regulation of MDSCs levels can provide a new approach for clinical immunotherapy of breast cancer.

Korkaya et al^26^ found that MDSCs were accumulated in bone marrow and spleen of mouse breast cancer model and promoted tumour proliferation and migration. In the present study, we observed that there was a significant accumulation of MDSCs in bone marrow and spleen in the 4T1 breast cancer model mice, and the numbers of *CD8 +* T lymphocytes in spleen were significantly decreased, indicating that there was a strong immunosuppressive microenvironment in breast cancer tumour. Exosome, a kind of extracellular vesicle with a diameter of 50‐200 nm nanometre, is the main component of paracrine. Exosomes contain a variety of active molecules such as active proteins, RNAs (lncRNA, microRNA, mRNA) and DNAs. These signal molecules are important for exosome‐mediated intercellular signal transduction.^17^, ^27^, ^28^ Exosomes secreted by tumour cells play an important role in tumour microenvironment,^29^ in which the exosomes exerted a strong immunosuppressive effect by blocking the differentiation of dendritic cells (DC). Tumour cell‐derived exosomes could inhibit the differentiation of bone marrow progenitor cells into DCs.^20^ Xiang et al^30^ found that *PGE2* and *TGF‐β* secreted by breast cancer cells could promote the differentiation of bone marrow cells into inflammatory MDSCs. Exosomes derived from human colorectal cancer and melanoma cells could inhibit the differentiation of peripheral blood monocytes into functional DCs and promote their differentiation into MDSCs.^31^ Zhang et al^21^ and Diao et al^32^ found that *HSP72* and *MYD88* Toll‐like receptor signals play a role in tumour exosome‐mediated MDSCs proliferation and tumour progression. In this paper, we also observed that the exosomes of 4T1 breast cancer cells promoted the differentiation of bone marrow cells into MDSCs. It has been reported that tumour exosomes could inhibit T‐cell activation and proliferation. In order to determine whether breast cancer cells‐derived exosomes have a function to differentiate bone marrow cells into MDSCs, the bone marrow cells with *CD3 +* T cells were co‐cultured with BCC‐Ex in vitro. The results showed that the bone marrow cells treated with BCC‐Ex inhibited the proliferation of T cells. In addition, our experiments in vivo showed that the exosomes of 4T1 breast cancer cells could induce the accumulation of MDSCs in bone marrow and decrease the numbers of T cells in spleen, resulting in an immunosuppressive microenvironment. It has been reported that the function of MDSCs was mainly mediated by enzymes such as *Arg‐1* and *iNOS*.^33^ Our study showed that the BCC‐Ex significantly increased the expressions of *Arg‐1* and *iNOS* in bone marrow cells, indicating that the exosomes could promote the production and proliferation of MDSCs.

It has been reported that tumour cells‐derived exosomes significantly down‐regulated the expressions of chemokine receptor *CXCR4* in mouse bone marrow cells, resulting in a decrease in their ability to migrate along with the *CXCL12*.^25^ Based on the observation that the BCC‐Ex decreased the expression of *CXCR4* in bone marrow cells, we speculated that the accumulation of MDSCs in bone marrow of 4T1 breast cancer tumour‐bearing mice might be due to the down‐regulation of *CXCR4* expression in bone marrow cells induced by BCC‐Ex, in which the bone marrow cells were not able to migrate to peripheral tissues for normal differentiation by inhibiting the *CXCL12‐CXCR4* signalling pathway and thus the cells were differentiated into MDSCs in bone marrow. The results that *CXCL12* partially reversed the effect of BCC‐Ex on the differentiation of bone marrow cells into MDSCs further supported our speculation. Therefore, the down‐regulation of *CXCR4* may be one of the factors promoting exosomes‐induced MDSCs production.^34^ BCC‐Ex can induce MDSCs differentiation by activating *STAT3* pathway and the release of *IL‐6*, *IL‐10* and other cytokines will further promote the activation and proliferation of MDSCs. Furthermore, we observed that BCC‐Ex inhibited the expressions of the apoptosis‐related protein Bax and the proliferation‐related protein PCNA, indicating that the BCC‐Ex promoted the differentiation of bone marrow cells into MDSCs by inhibiting the proliferation and apoptosis of bone marrow cells.

Studies shown that MDSCs were able to promote immune tolerance and the progression and metastasis of breast cancer by providing a immunosuppressive microenvironment. Although we demonstrated that BCC‐Ex differentiated the bone marrow cells into MDSCs in vitro, we have not directly verified the effect of BCC‐Ex on the proliferation and metastasis of breast cancer at the animal level. In the follow‐up study, we will investigate the effects of *STAT3* signal pathway and *CXCR4* on the development and metastasis of breast cancer at the animal level. In addition, it is necessary to further study the role of *STAT3* signal pathway in the BCC‐Ex‐induced MDSCs.

## 5 CONCLUSIONS

In summary, we demonstrated that BCC‐Ex differentiated the bone marrow cells into MDSDs and inhibited the proliferation of T lymphocytes in vitro and in vivo and the possible mechanisms may be related to BCC‐Ex‐mediated the activation of *STAT3* signal pathway and down‐regulation of the expression of chemokine receptor *CXCR4*. Obviously, elucidation of the mechanism of BCC‐Ex on MDSCs generation should provide an insight into the development and immunotherapy of breast cancer.

## CONFLICT OF INTEREST

The authors have declared that no competing interests exist.

## AUTHOR CONTRIBUTIONS

Quan‐Wen Liu: Conceptualization‐Equal, Data curation‐Equal, Formal analysis‐Equal, Funding acquisition‐Equal, Methodology‐Equal and Project administration‐Equal. Yong Chen, Jing‐Yuan Li and Ling Xiao: Data curation‐Equal and Investigation‐Equal. Wen‐Jie Zhang: Formal analysis‐Equal. Jia‐Le Zhao: Investigation‐Equal. Hao‐Cheng Gu: Data curation‐Equal. Han‐You Wu: Software‐Equal. Guo‐Si‐Lang Zuo: Methodology‐Equal. Ke‐Yu Deng: Funding acquisition‐Equal and Project administration‐Equal. Hong‐Bo Xin: Conceptualization‐Equal, Funding acquisition‐Equal and Project administration‐Equal.

## Supporting information

Figure S1

Figure S2

## Data Availability

The data that support the finding of this study are available from the corresponding author upon reasonable request.
