# Supplementary figures and images for: Bone marrow cells are differentiated into MDSCs by BCC‐Ex through down‐regulating the expression of CXCR4 and activating STAT3 signalling pathway
Source: J Cell Mol Med. 2021 May 6;25(12):5497–510. doi: 10.1111/jcmm.16559 (PMC8184685; doi:10.1111/jcmm.16559)

**Supplemental Figure 1. Representative images of tumor from 4T1 breast cancer model mice**

**
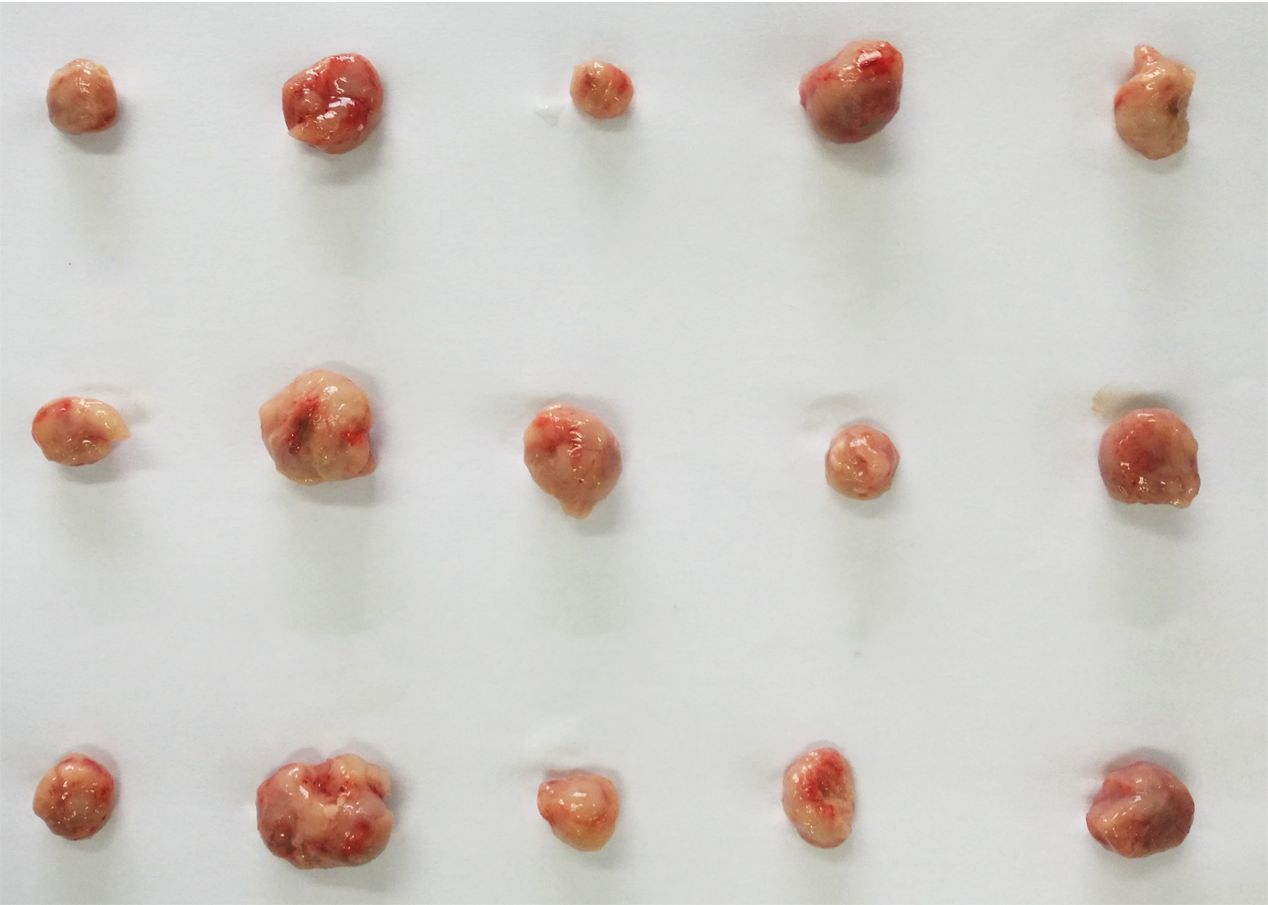
**

Supplement: Supplementary file 1 — Figure S1 [file JCMM-25-5497-s002.docx]
