# Supplementary material for: Bone marrow cells are differentiated into MDSCs by BCC‐Ex through down‐regulating the expression of CXCR4 and activating STAT3 signalling pathway
Source: J Cell Mol Med. 2021 May 6;25(12):5497–510. doi: 10.1111/jcmm.16559 (PMC8184685; doi:10.1111/jcmm.16559)

**Supplemental Figure 2. The amount changes of mMDSCs and gMDSCs in bone marrow and spleen of E0771 and MAD-MB-231 tumor-bearing mice and normal control mice.** (A) Detect the amounts of mMDSCs and gMDSCs in bone marrow and spleen in the normal mice and tumor-bearing mice with breast cancer cell lines E0771 and MAD-MB-231.（B-C）Quantitative analysis of the amounts of mMDSCs and gMDSCs in bone marrow and spleen in the normal mice and tumor-bearing mice with breast cancer cell lines E0771 and MAD-MB-231 in figure A (n=3).


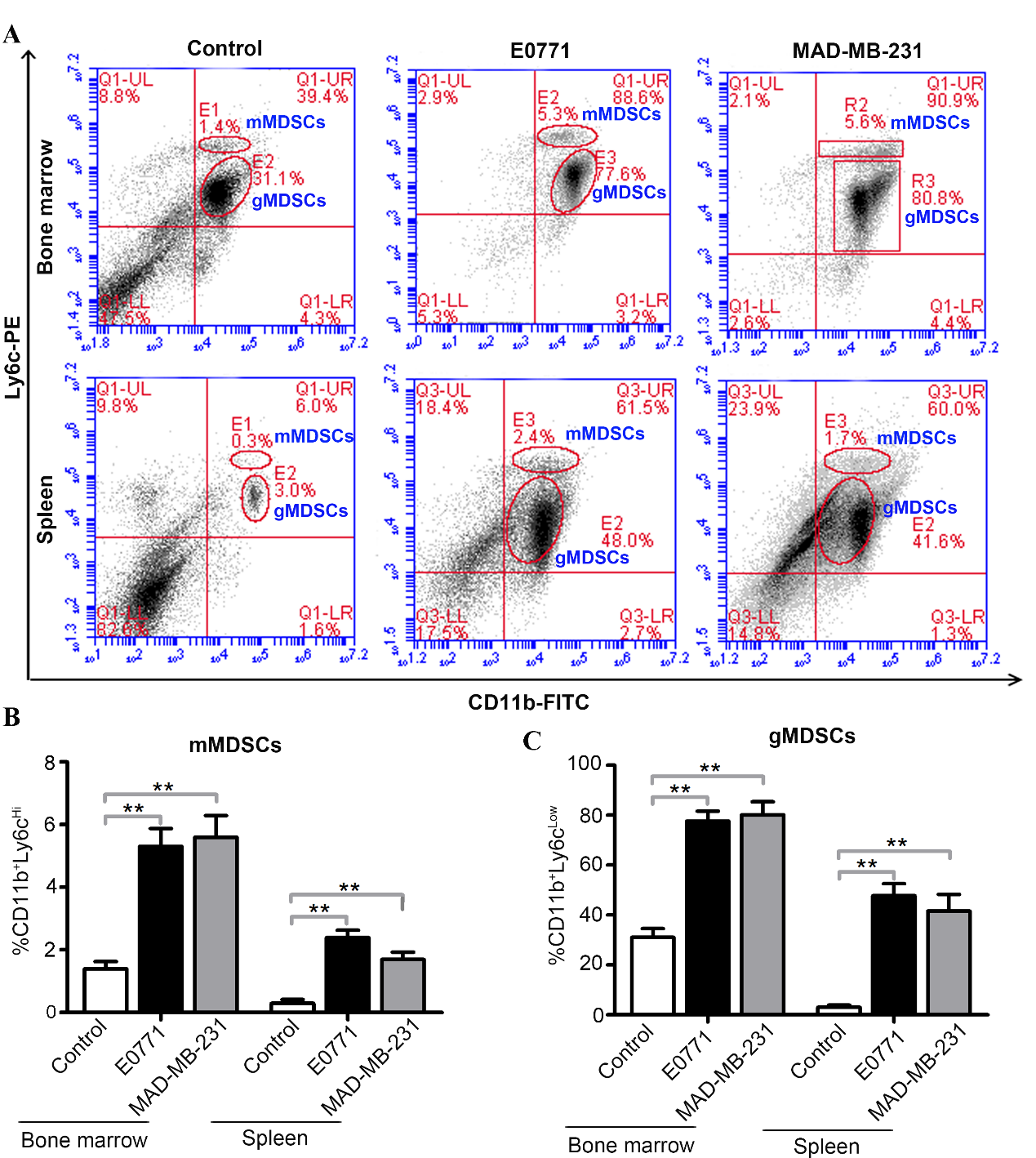

Supplement: Supplementary file 2 — Figure S2 [file JCMM-25-5497-s001.docx]
